# Case report of sigmoid colon perforation and colocutaneous fistula due to retropubic midurethral sling placement for stress urinary incontinence

**DOI:** 10.1186/s12894-020-00600-x

**Published:** 2020-03-20

**Authors:** Xiao Huang, Hai Jiang, Liping Xie

**Affiliations:** Department of Urology, The First Affiliated Hospital, School of Medicine, Zhejiang University, No. 79 QingChun Road, Hangzhou, 310003 Zhejiang Province PR China

**Keywords:** Stress urinary incontinence, Tension-free vaginal tape, Sigmoid colon perforation

## Abstract

**Background:**

Sigmoid bowel perforation is a very rare and serious complication of the retropubic tension-free vaginal tape (TVT) procedure for female stress urinary incontinence. The complication can be avoided with the use of the correct manipulation technique.

**Case presentation:**

A 75-year-old female patient underwent a retropubic TVT procedure in the local hospital for the treatment of stress urinary incontinence. The procedure was smooth. Two weeks after surgery, the patient began to complain of fever and bloody, purulent discharge from the left suprapubic skin wound. During a 4-month period after surgery, she was admitted to the local hospital 4 times for similar infection symptoms. The infections were temporarily controlled with antibiotic administration. The reason for the refractory infection of the left suprapubic skin wound was not identified until a foreign TVT mesh was found in the sigmoid colon via a colonoscopy. We diagnosed that the TVT mesh caused a sigmoid colon perforation that led to colocutaneous fistula. An exploratory laparotomy revealed that the TVT tape perforated into and out of the sigmoid colon. An 8-cm long left part of mesh was removed. Two ruptures of sigmoid colon were mended without the need for bowel resection. At the 4-years follow-up after laparotomy, the patient was doing well and still continent.

**Conclusions:**

Urologists and gynecologists should be aware of the possibility of colon bowel injury in SUI patients with prior sling surgeries. Patient having recurrent suprapubic cutaneous infection may have high degree of suspicion of colon injury after TVT sling. The passage of the retropubic space procedure should be slow and always along the pubic bone according to the anatomy.

## Background

Stress urinary incontinence (SUI) is a major urological/gynecological problem. As many as 25% of women older than 20 years have urinary incontinence [1]. The retropubic tension-free vaginal tape (TVT) procedure has become the standard of care for SUI with demonstrated efficacy and limited complications since its introduction in 1996 by Ulmsten et al. [2]. However, complications do occur during or after the procedure, ranging from bladder perforation (3.8%), urethral lesion (0.07%) to intestinal injury (0.03–0.7%) [3, 4]. Bowel injuries have rarely been reported in the literature, with most reported cases involving the small intestine becoming clinically apparent in perioperative period after the procedure [5, 6]. In some cases, bowel perforations may be found very late ranging from several months to years [7–9], especially in cecum [10] and sigmoid colon [11], which is very similar to what was found in our case but that occurred 7 years after surgery. We recently experienced a case of sigmoid colon perforation and colocutaneous fistula 2 weeks after the TVT tape procedure was performed.

## Case presentation

**Fig. 1:**
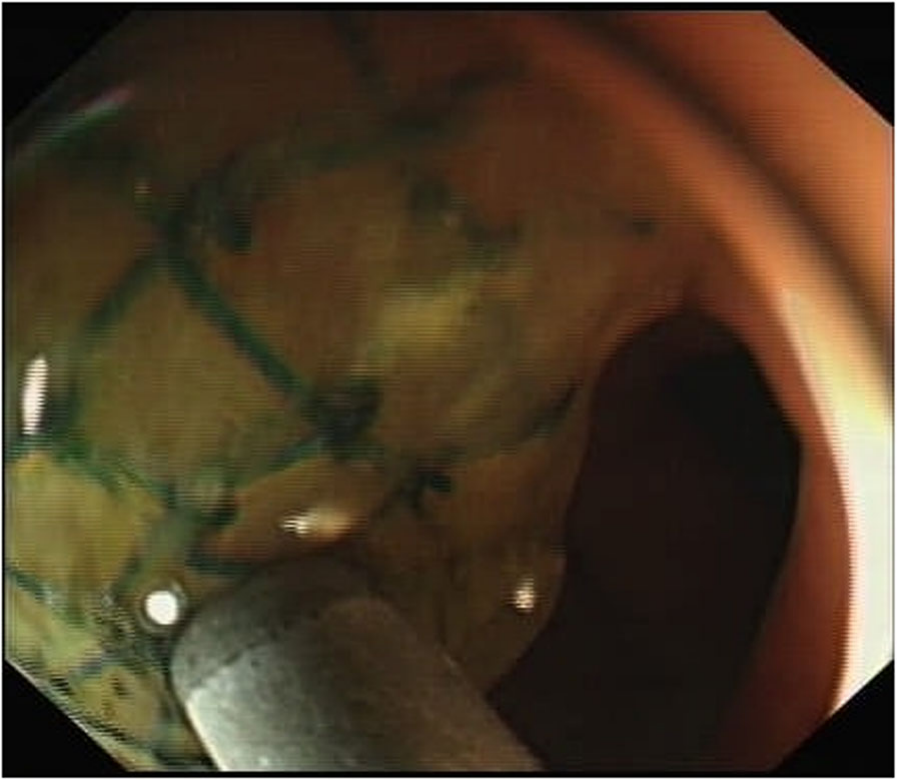
A foreign mesh was revealed in the sigmoid colon 30 cm from the anal orifice via colonoscopy

**Fig. 2:**
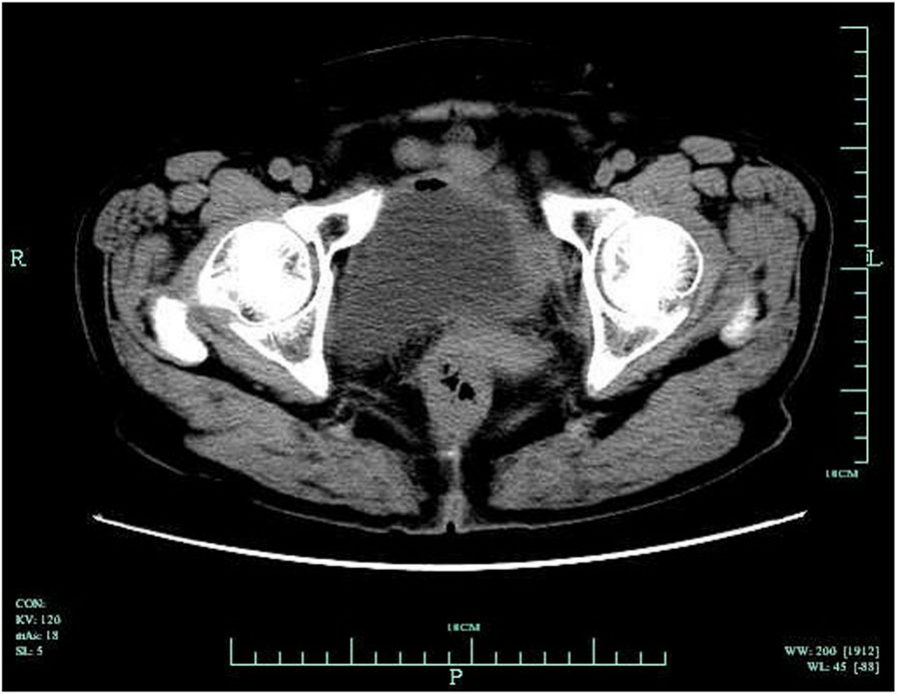
Computed tomography showing the thickened local left bladder wall and the internal iliac muscle

**Fig. 3:**
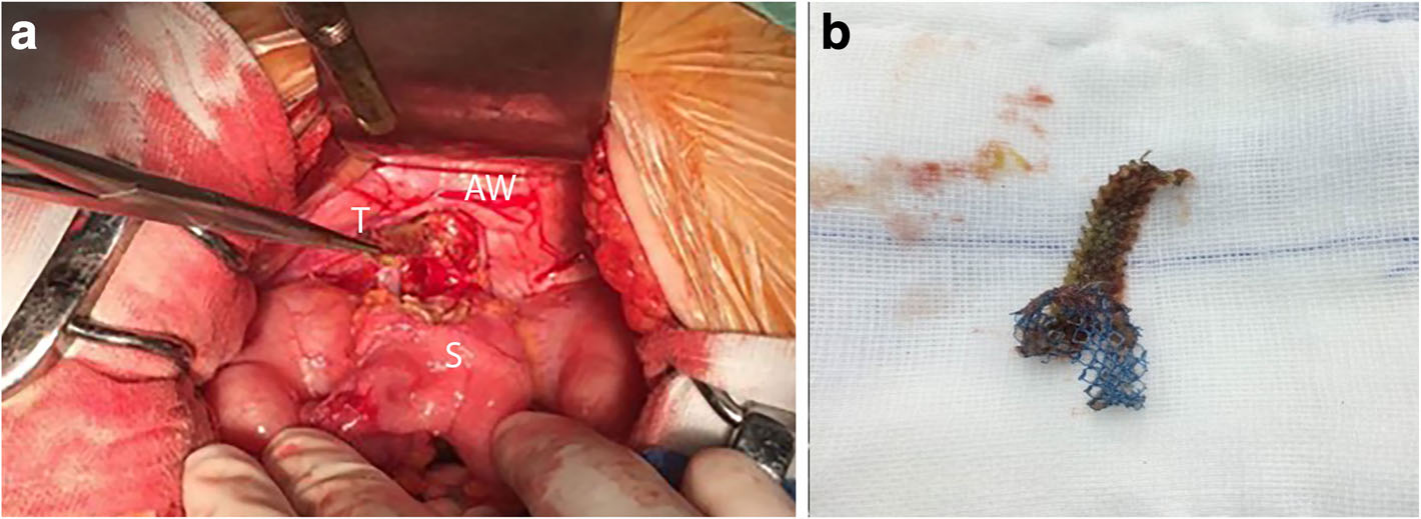
A The sigmoid colon was perforated and fixed to the left abdominal wall (AW, abdominal wall; T, tape; S, sigmoid) by an 8-cm long TVT tape. b The foreign tape was removed with exploratory laparotomy. The brown part of the tape was in the sigmoid cavity, and the blue part was out of the sigmoid cavity

A 75-year-old woman was referred to our center from local hospital with a history of a retropubic TVT sling (TVT-Exact; Gynecare) procedure performed 4 months ago due to SUI. The procedure was smooth. The patient began to complain of fever (with body temperature from 37.8~38.6 °C) and bloody, purulent discharge from left suprapubic skin wound 2 weeks after the TVT surgery. No nausea and vomiting. No peritoneal irritation sign. The volume of purulent discharge from skin fistula was 5-10 ml per day. Laboratory data showed elevated levels of white blood cells of 1.2 × 10^9^/L and C-reactive protein (CRP) of 20.85 mg/L. The patient had been admitted to the local hospital 4 times because of similar symptoms in those 4 months after surgery. After the administration of kanamycin or quinolones antibiotics for about 3–7 days, the patient’s symptoms were resolved completely between episodes. The left suprapubic infection and the tape exit wound purulent discharge was recurrent and could be temporarily controlled after antibiotic administration. Cystoscopy showed no bladder injury. The reason for recurrent left suprapubic wound infection was finally identified by a colonoscopy in local hospital, which showed a foreign TVT tape mesh in the sigmoid colon cavity approximately 30 cm from the anal orifice (Fig. 1). The patient was then transferred to our center for further treatment. The patient denied urinary symptoms and was continent. She had no abdominal surgery history before this TVT procedure. Her medical history was significant for diabetes mellitus II, and the blood glucose levels were well controlled with insulin and acarbose. Upon physical examination, the patient’s vital signs were normal. The abdomen was flat with no signs of peritonitis. There was a 1 × 1-cm red swollen area at the left suprapubic skin with purulent secretion. Abdominal computed tomography (CT) showed a thickened left bladder wall and internal iliac muscle. No abscess was found in the pelvic cavity (Fig. 2). We diagnosed that the TVT mesh caused a sigmoid colon perforation that led to colocutaneous fistula. An exploratory laparotomy was performed through an incision in the midline of the lower abdomen. The TVT tape was found to have perforated the sigmoid bowel in an in-and-out fashion before exiting at the suprapubic skin incision. The sigmoid colon was fixed to the abdominal wall by the tape. After the two sides of the tape were mobilized and cut, an 8 cm-long whole left part of the mesh was entirely removed from the sigmoid colon and the retropubic space (Fig. 3). The two ruptures of the sigmoid colon were mended with intermittent sutures. No resection of the bowel was needed. The patient recovered well from the procedure and was discharge 2 weeks after the surgery. Four years following the laparotomy and partial tape removal, the patient was doing well and still continent.

## Discussion and conclusions

Although the retropubic TVT sling procedure has been demonstrated to be a safe and effective surgery for SUI, there have been reports of complications, such as hematoma formation, urinary retention and mesh erosion [3, 4]. Bowel perforation which is reported in only 0.03%~ 0.7% of sling surgeries, is a very rare complication of TVT type sling procedures [4]. Most cases have been reported in patients with a previous history of abdominal or pelvic surgery [7–9]. However, our patient had no history of abdominal or pelvic surgery. When bowel injury is noted, it typically involves displacement of the mesh into the abdominal cavity with resulting perforation of the bowel.

Bowel injuries involving the small intestine usually have acute symptoms such as fever, nausea, vomiting, abdominal pain, distention and diminished bowel sounds because of peritonitis and bowel obstruction and can be diagnosed early [5, 6]. Whereas bowel injuries involving colon [10, 11], it may be found very late ranging from several months to years. Our patient had none of the general symptoms described above. But she mainly had a local suprapubic skin infection, similar to the case reported in the literature [11], which occurred 7 years after surgery. In our case, the sigmoid colon perforation occurred during the surgery. But it took 4 months for surgeons in the local hospital to identify the bowel injury. Therefore, a colonoscopy examination may be needed immediately if a recurrent local infection cannot be explained after the TVT procedure. In our case, it appeared that the intraperitoneal placement of the TVT tape had just perforated into the sigmoid colon and fixed the colon segment to the abdominal wall. The subsequent infection occurred between the involved sigmoid colon and subcutaneous tissue along the tape, so the patient had no symptoms of peritonitis or bowel obstruction but only a local skin fistula formation.

The reason for this complication may be the surgeon’s lack of experience. According to the standard TVT technique, the skin site of the tape exit should be 2 cm from the midline, and the passage of the retropubic space procedure should be slow and always along the pubic bone according to the anatomy. The force and direction of the puncture should be well controlled, so that the passage of the needle is not too fast accidently entering the abdominal cavity. Because of the blind passage technique, bowel perforations may not be immediately recognized during the surgery. The blind passage technique limits the utility of observation, so a close observation of trainees is strongly recommended. Before performing the TVT procedure alone, all trainees should have sufficient practice under the observation of an experienced surgeon. Clinical suspicion of bowel injury must be maintained for any unexplained postoperative abdominal symptoms. Patient having recurrent suprapubic subcutaneous infection may have high degree of suspicion of colon injury after TVT sling. Colonoscopy is needed to verify the suspicion. After bowel injury is confirmed, an open or laparoscopic exploratory laparotomy [11] with excision of the mesh and repair of the bowel should be performed promptly.

## Data Availability

The datasets used and analysed during the current study are available from the corresponding author on reasonable request.

## Abbreviations

SUI: Stress urinary incontinence
TVT: Tension-free vaginal tape
CT: Computed tomography
CRP: C-reactive protein
